# Complications des prises en charge chirurgicales des abdomens aigus non traumatiques d'origine digestive à l'hôpital central de Yaoundé, Cameroun (novembre 2019 - juillet 2020)

**DOI:** 10.48327/mtsi.2021.99

**Published:** 2021-11-26

**Authors:** Guy Aristide BANG, Georges BWELLE MOTO, Joseph Cyrille CHOPKENG NGOUMFE, Yannick Mahamat EKANI BOUKAR, Fabrice TIENTCHEU TIM, Eric Patrick SAVOM, Arthur ESSOMBA

**Affiliations:** 1 Département de chirurgie et spécialités, Faculté de médecine et des sciences biomédicales de l'Université de Yaoundé I, Cameroun; 2 Service de chirurgie viscérale et digestive, Hôpital central de Yaoundé, Cameroun

**Keywords:** Urgence chirurgicale digestive, Abdomen aigu chirurgical, Morbidité, Mortalité, Facteurs de risque, Postopératoire, Péritonite aiguë généralisée, Appendicite aiguë, Occlusion intestinale, Suivi postopératoire, Hôpital, Yaoundé, Cameroun, Afrique subsaharienne, Digestive surgical emergency, Digestive acute abdomen, Morbidity, Mortality, Risk factors, Postoperative course, Acute generalized peritonitis, Acute appendicitis, Intestinal obstruction, Post-operative follow-up, hospital, Yaounde, Cameroon, Sub-Saharan Africa

## Abstract

**Objectif:**

Les abdomens aigus chirurgicaux non traumatiques d'origine digestive demeurent un motif fréquent d'admission aux urgences en Afrique. Nous avons entrepris ce travail dans le but d’étudier la morbi-mortalité postopératoire de ces patients au Cameroun, un pays d'Afrique centrale en voie de développement.

**Patients et méthodologie:**

Il s'agissait d'une étude transversale analytique avec recueil prospectif de données, sur une période de huit mois (novembre 2019 à juillet 2020), à l'hôpital central de Yaoundé (Cameroun). Il s'agit d'une structure sanitaire publique de deuxième catégorie (intermédiaire) dans la pyramide sanitaire du Cameroun, accueillant majoritairement des patients sans assurance maladie. Ont été inclus, tous les patients opérés pour un abdomen aigu chirurgical digestif non traumatique. Le suivi des patients se faisait jusqu’à la 12^e^ semaine postopératoire. La régression univariée de Cox a été utilisée pour déterminer les facteurs associés à la survenue de complications postopératoires. Le seuil de significativité retenu était 0,05.

**Résultats:**

Nous avons colligé 120 patients, représentant 14,6 % de toutes les urgences chirurgicales. L’âge moyen des patients était de 37,6 ± 13,5 ans. Quatre-vingts (66,7 %) étaient de sexe masculin, soit un sex-ratio de 2. Les deux principaux diagnostics préopératoires étaient la péritonite aiguë généralisée (n = 58 soit 48,3 %) et l'occlusion intestinale (n = 38 soit 31,7 %). Les deux principales étiologies des abdomens aigus étaient la perforation d'ulcère gastroduodénal (n = 35) et l'appendicite aiguë (n = 24). Le délai moyen de consultation était de 1,9 jours et en moyenne 36,8 heures s’écoulaient entre le diagnostic et l'intervention chirurgicale. En postopératoire les taux de morbidité et de mortalité étaient respectivement de 33,3 et 10 %. Les complications postopératoires étaient majoritairement mineures selon la classification de Clavien-Dindo soit 21 (33,8 %) de grade I et 12 (19,3 %) de grade II. La principale cause de mortalité était la septicémie (8 cas sur 12). Nous avons identifié sept facteurs statistiquement associés à un risque accru de survenue de complications en postopératoire dont trois modifiables: un délai de consultation supérieur à 72 heures (p = 0,02), un délai entre le diagnostic et l'intervention chirurgicale supérieur à 48 heures (p = 0,01) et une durée d'intervention supérieure à deux heures (p = 0,05).

**Conclusion:**

Dans notre contexte, les résultats de la prise en charge chirurgicale des abdomens aigus non traumatiques d'origine digestive sont marqués par une morbi-mortalité élevée. Les pistes de solution sont: l'organisation de campagnes de sensibilisation des populations à une consultation rapide en cas de douleurs abdominales aiguës, la mise sur pied d'une couverture sanitaire universelle ainsi que l'amélioration du plateau technique.

## Abstract (en)

**Objective:**

Acute non-traumatic digestive surgical emergencies are a frequent cause of emergency in Africa. We undertook this study to investigate the morbidity and mortality of these patients in Cameroon, a developing country in Central Africa.

**Patients and methodology:**

This was an analytical cross-sectional study with prospective data collection, over a period of eight months (November 2019 to July 2020), at the Yaoundé central hospital (Cameroon). The latter is a second category (intermediate) public health facility in the Cameroon health pyramid, mainly welcoming patients without health insurance. All patients operated on for an acute non-traumatic digestive abdomen were included. The patients were followed up until the 12^th^ postoperative week. We used Cox univariate regression to determine factors associated with the occurrence of postoperative complications. The significance threshold retained was 0.05.

**Results:**

We collected 120 patients, representing 14.6% of all surgical emergencies. The mean age of the patients was 37.6 ± 13.5 years. Eighty (66.7%) were male with a sex ratio of 2. The two main preoperative diagnoses were acute generalized peritonitis (n = 58 or 48.3%) and intestinal obstruction (n = 38 or 31.7%). The two main etiologies were peptic ulcer perforation (n = 35) and acute appendicitis (n = 24). The delay between the onset of symptoms and consultation was 1.9 day and an average of 36.8 hours elapsed between diagnosis and surgery. During postoperative time the morbidity and mortality rates were 33.3 and 10%, respectively. Postoperative complications were mostly minor according to the Clavien-Dindo classification, with 21 cases of grade I (33.8%) and 12 cases of grade II (19.3%). The main cause of death was sepsis (8 out of 12 cases). We identified seven factors significantly associated with an increased risk of postoperative complications among whom three were modifiable: The consultation delay greater than 72h (p = 0.02), the time between diagnosis and the surgical intervention greater than 48h (p = 0.01) and the operating time greater than 2h (p = 0.05).

**Conclusion:**

In our context, the results of the surgical management of acute non-traumatic abdomens of digestive origin are marked by high morbidity and mortality. The possible solutions are: the organization of public awareness campaigns to prompt rapid consultation in the event of acute abdominal pain, the establishment of universal health coverage as well as the improvement of technical platforms.

## Introduction

Les abdomens aigus chirurgicaux d'origine digestive demeurent un motif fréquent d'admission aux urgences en Afrique. Ils se définissent comme des affections digestives qui, faute d'une intervention chirurgicale en urgence font succomber les patients en quelques heures ou peu de jours [22].

La période postopératoire de ces patients est souvent émaillée d'une morbi-mortalité non négligeable [25]. Ces complications postopératoires sont plus fréquentes en milieu défavorisé [4]. Certains travaux rapportent un taux de morbidité de 50,6 % et un taux de mortalité de 8,2 % [15, 23]. L'infection du site opératoire (ISO) est la complication postopératoire la plus fréquente [2, 18]. L’étude des résultats de la prise en charge des urgences chirurgicales digestives peut permettre d'identifier les causes de la morbi-mortalité postopératoire pour en réduire l'incidence. Les données sur cette problématique demeurent toutefois parcellaires dans notre environnement, d'où cette étude.

## Patients et méthodes

Il s'agissait d'une étude descriptive prospective analytique, qui s'est déroulée à l'Hôpital central de Yaoundé.

La pyramide sanitaire du Cameroun répartit les formations sanitaires en trois catégories, la 1re catégorie étant la plus élevée. L'hôpital central de Yaoundé est la seule structure sanitaire de deuxième catégorie de la ville de Yaoundé, qui compte par ailleurs trois hôpitaux de première catégorie et près d'une cinquantaine de structures sanitaires de troisième catégorie. C'est un hôpital universitaire de type pavillonnaire, qui accueille majoritairement des patients sans couverture sanitaire; ces patients sont issus non seulement de la ville de Yaoundé, mais sont aussi adressés par des structures sanitaires de niveaux trois des villes environnantes. Quasiment toutes les spécialités chirurgicales y sont présentes.

L’étude s'est déroulée dans deux services de cet hôpital: le bloc des urgences chirurgicales et le service de chirurgie viscérale et digestive. Le bloc des urgences chirurgicales est le service dédié à la prise en charge de toutes les urgences chirurgicales admises à l'hôpital central de Yaoundé. Il ne dispose que de deux salles d'interventions chirurgicales; ainsi, certaines urgences chirurgicales digestives sont régulièrement prises en charge dans le service de chirurgie viscérale et digestive lorsque le bloc des urgences chirurgicales est saturé.

Sur une période allant de novembre 2019 à juillet 2020 (soit huit mois), nous avons colligé en prospectif, tous les patients opérés pour un abdomen aigu chirurgical non traumatique d'origine digestive. Les autres causes d'abdomen aigu non traumatiques (gynécologiques ou urologiques) et traumatiques n’étaient pas inclues. Le suivi des patients s'est poursuivi jusqu’à la douzième semaine post-opératoire.

Les variables étudiées étaient: les données sociodémographiques, l’étiologie de l'abdomen aigu, le score de l'American Society of Anesthesiologists (ASA) [19], le type de chirurgie selon Altemeier [1], le geste opératoire, la durée de l'intervention, et la morbi-mortalité postopératoire sur trois mois. De toutes ces variables, seule la survenue d'une complication postopératoire a été considérée comme dépendante. Les complications postopératoires étaient répertoriées selon la classification de Clavien-Dindo [8].

Les données ont été enregistrées à l'aide du logiciel Microsoft Excel 2016, et analysées à l'aide du logiciel Epi-info 3.5.4. Les variables quantitatives ont été exprimées sous forme de moyenne ± écart-type et les variables qualitatives résumées sous forme de proportion. La régression logistique de Cox a été utilisée pour identifier les facteurs associés à la survenue des complications post-opératoires avec leur rapport de risque (HR), la différence étant considérée comme statistiquement significative pour une valeur de p ≤ 0,05.

Notre protocole d’étude a reçu la clairance du Comité d’éthique de l'Université de Douala sous le numéro 1749 CEI-Udo/06/2014/T. Nous avons obtenu l'accord du directeur de l'hôpital central de Yaoundé avant le début de l’étude, ainsi que le consentement éclairé de chaque patient avant toute inclusion dans ce travail.

## Résultats

Durant la période d’étude, 822 patients ont été admis pour prise en charge d'une urgence chirurgicale. Parmi-eux, 120 (14,6 %) remplissaient nos critères d'inclusion, soit une incidence hospitalière mensuelle de 15 cas. Quatre-vingts d'entre eux étaient de sexe masculin, soit un sex-ratio H/F de 2. L’âge moyen des patients était de 37,6 ± 13,5 ans. La tranche d’âge la plus représentée était celle comprise entre 15 et 25 ans. Le tableau I résume les caractéristiques sociodémographiques des patients.

**Tableau I:** Caractéristiques sociodémographiques des patients Patients’ sociode mographic features

Caractéristique		**n**	**%**
Sexe	masculin	80	67
	féminin	40	33
Tranches d’âge	15-25	36	30
	26-35	34	28,3
	36-45	16	13,3
	46-55	14	11,7
	56-65	6	5
	> 65	14	11,7
Profession	fonctionnaire	16	13,3
	commerçant	22	18,3
	ménagère	6	5
	élève/étudiant	36	30
	ouvrier/artisan/paysan	28	23,4
	Retraité	12	10

Les étiologies des abdomens aigus (Tableau II) étaient par ordre décroissant: péritonite aiguë généralisée (n = 58 soit 48,3 %), occlusion intestinale (n = 38 soit 31,7 %) et appendicite aiguë non compliquée (n = 24 soit 20 %).

**Tableau II:** Étiologies des abdomens aigus Etiologies of acute abdomens

Diagnostic	n	%
**Péritonite aiguë généralisée**	58	48,3
perforation gastrique	35	29,2
origine appendiculaire	6	5
perforation typhique	17	14,2
**Occlusion intestinale aiguë**	38	31,7
tumorale	18	15
hernie étranglée	8	6,6
sur brides	7	5,8
volvulus	5	4,2
**Appendicite aiguë non compliquée**	24	20

Le délai moyen entre le début des symptômes et la consultation était de 1,9 ± 1,4 jour avec des extrêmes allant de 0 à 7 jours. La majorité des patients (67,5 %) étaient ASA III. Selon la classification Altemeier, la chirurgie était le plus souvent de type sale (n = 58 soit 48,3 %). Le tableau III résume les classifications ASA et Altemeier des patients.

**Tableau III:** Classifications ASA et ALTEMEIER des patients Patients’ ASA and ALTEMEIER classifications

ITEM		n	%
ASA
	ASA II	24	20
	ASA III	81	67,5
	ASA IV	15	12,5
	ASA V	0	0
ALTEMEIER^*^			
	propre contaminée	38	31,7
	contaminée	24	20
	sale ou infectée	58	48,3

* Classe de contamination des interventions chirurgicales [1]

Le délai moyen entre le diagnostic et le début de l'intervention chirurgicale était de 36,8 ± 8,4 h avec des extrêmes allant de 2 h à 321 h. Le geste opératoire le plus fréquent était la suture d'une perforation d'organe creux (n = 38). Le tableau IV présente les gestes opératoires effectués.

**Tableau IV:** Principaux gestes opératoires effectués Main surgical procedures performed

	n	%
Appendicectomie	38	31,7
Herniorraphie	8	6,7
Résection/anastomose	28	23,3
Suture d'une perforation	38	31,7
Adhésiolyse	7	5,8
Stomie		
Colostomie	10	8,3
Iléostomie	5	4,2

La durée moyenne de l'intervention était de 109,8 ± 25,5 minutes avec des extrêmes compris entre 50 minutes et 195 minutes.

**Figure 1:**
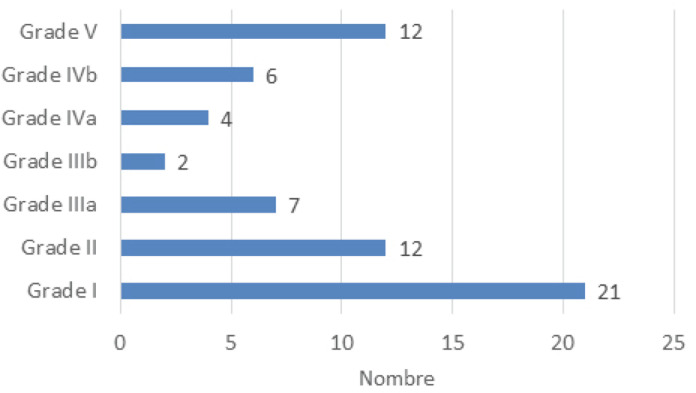
Répartition des complications selon la classification de Clavien-Dindo Complications according to the Clavien-Dindo's classification

En postopératoire, 40 patients ont présenté des complications, soit un taux de morbidité de 33,3 %. Sur les 62 complications enregistrées, la majorité étaient mineures selon la classification de Clavien-Dindo (Fig. 1), soit 21 (33,8 %) de grade I et 12 (19,3 %) de grade II.

**Figure 2:**
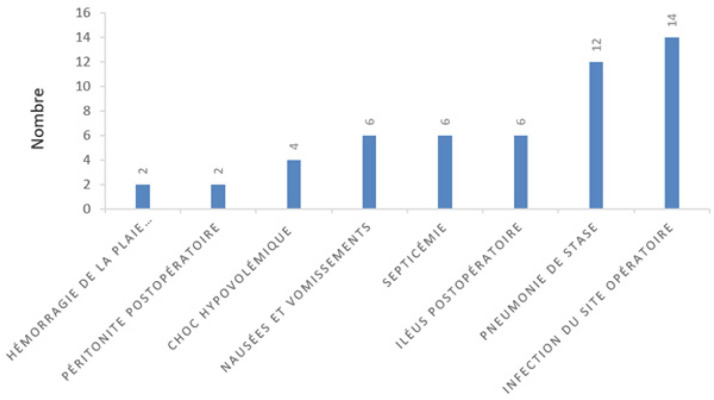
Complications postopératoires observées Postoperative complications

En analyse univariée de Cox, les facteurs statistiquement associés à la survenue des complications post opératoires étaient: la tranche d’âge > 65 ans (HR = 0,5 et p = 0,003), un délai de consultation supérieur à 72 h (HR = 0,1 et p = 0,02), la classe ASA III (HR = 0,2 et p = 0,0002), un délai d'intervention supérieur à 48h (HR = 0,9 et p = 0,01), le diagnostic de péritonite (HR = 0,5 et p = 0,03), une durée d'intervention supérieure à 2h (HR = 1,01 et p = 0,05) et la chirurgie sale selon Altemeier (HR = 2,4 et p = 0,003).

Douze décès ont été enregistrés, soit un taux de létalité de 10 %. Les causes de ces décès étaient: une septicémie (n = 8 soit 66,7 %) et un choc hypovolémique (n = 4 soit 33,3 %). Les patients décédés avaient été opérés, soit pour une péritonite (n = 6), soit pour une occlusion intestinale (n = 6).

Le coût moyen global de la prise en charge de ces patients était de 357 450 ± 121 575 F CFA (544,93 ± 188,34 €).

## Discussion

Les limites de notre étude sont liées à son caractère monocentrique, ainsi que sa durée et son échantillon relativement faibles; en effet, notre bassin de recrutement est large avec de nombreuses structures sanitaires autour de notre hôpital. Ceci nous oblige à rester modestes pour la généralisation de nos résultats. Toutefois, le recueil prospectif des données en est une force, la plupart des autres études africaines sur le sujet étant rétrospectives [2,11,15,16,23].

Avec une incidence hospitalière mensuelle de 15 cas, cette étude confirme que l'abdomen aigu chirurgical digestif occupe une place importante dans l'activité d'un chirurgien général/viscéral en Afrique [11, 16]: une étude au Niger en 2016 rapportait une incidence hospitalière mensuelle de 25,9 cas [17].

L'adulte jeune était le plus représenté dans notre cohorte, comme dans d'autres études d'Afrique subsaharienne [11,14,17,23]. On peut donc penser que l'impact socio-économique des urgences chirurgicales digestives est important, quand on sait qu'il s'agit de la tranche d’âge la plus active économiquement de la population.

Les péritonites aiguës généralisées par perforation gastroduodénale étaient la principale étiologie d'abdomens aigus chez nos patients. Malgré les progrès enregistrés ces dernières années dans la compréhension de la physiopathologie et la prise en charge de l'ulcère gastroduodénal [3, 13], force est de constater que cette affection demeure un problème de santé important en Afrique [24, 26, 27] avec un faible accès des populations à l'endoscopie digestive haute et aux inhibiteurs de la pompe à protons. Il n'est donc pas surprenant que ses complications aiguës, telle la perforation, soient encore fréquentes. L'appendicite aiguë était la seconde étiologie des abdomens aigus dans notre série. S'il est de plus en plus recommandé dans certaines conditions de faire le traitement médical des appendicites aiguës non compliquées [9], le traitement de cette affection demeure encore principalement chirurgical.

Si dans certaines études subsahariennes, la perforation typhique demeure la principale étiologie des péritonites aiguës généralisées [5, 10], on note toutefois une tendance à la régression de la fréquence de cette étiologie [4, 7, 23]. La vaccination, l'amélioration des conditions d'hygiène alimentaire et de salubrité, ainsi que l'accès plus facile aux antibiotiques sont des pistes de d'explication.

Le taux de morbidité postopératoire dans notre étude était élevé, de l'ordre de 33,3 %, ceci en accord avec de précédents travaux qui retrouvaient une morbidité variant entre 16,6 et 50,6 % [17, 23, 25]. Nous avons retrouvé une létalité mortalité globale de 10 %. De précédentes études dans notre contexte retrouvaient une létalité comprise entre 5,8 et 15,2 % [4, 23].

Nous avons identifié sept facteurs associés statistiquement à un risque accru de survenue de complications postopératoires. Trois d'entre eux ont particulièrement retenu notre attention car étant, à notre avis, modifiables.

Le premier de ces facteurs est le délai (supérieur à 72 heures) entre le début des symptômes et la consultation. Si le délai moyen de consultation était relativement court dans notre étude (1,9 jour), près de 67,5 % des patients présentaient déjà une perturbation sévère d'une grande fonction (classés ASA 3), soulignant ainsi un retard diagnostique important. Ceci pourrait s'expliquer par le parcours thérapeutique complexe des patients africains avant leur arrivée à l'hôpital [6, 20]. En effet, la survenue d'une maladie, et en l'occurrence d'une douleur abdominale aiguë, est souvent perçue comme la manifestation d'un mauvais sort (empoisonnement mystique). Le premier réflexe des patients est alors de se tourner vers les guérisseurs traditionnels et/ou les prières, la consultation à l'hôpital se faisant en dernier recours, à un stade avancé [12, 20, 21]. Des études ont montré que certains patients ayant une péritonite aiguë généralisée, étaient vus en consultation 13 jours après l'apparition des premiers symptômes [4]. La sensibilisation des patients, via des relais communautaires ou des campagnes publicitaires, les incitant à consulter rapidement dans une structure hospitalière en cas de douleur abdominale peut contribuer à réduire le retard diagnostique.

Le second facteur était le retard de la prise en charge chirurgicale (supérieur à 48 heures). Notre étude a retrouvé un délai moyen entre le diagnostic et l'intervention chirurgicale de 36,8 heures. Du fait de l'absence d'un système de couverture sanitaire universelle dans notre pays, les patients et leurs familles doivent payer eux-mêmes toutes les prescriptions médicales et le coût de l'intervention chirurgicale pour que celle-ci se réalise. Une fois les ordonnances remises, le temps de collecte des contributions financières des différents membres de la famille/communauté pour payer les soins, peut être long. Dans un pays où le revenu moyen mensuel est de 33 000 F CFA (55 €), le coût global moyen de prise en charge de 357 450 F CFA (545 €) est largement au-dessus des moyens financiers de la majorité de la population. L’élaboration d'une politique nationale efficiente de financement des soins de santé est donc un impératif. Une autre explication du retard de prise en charge est le temps mis pour réunir le matériel de chirurgie/anesthésie, une fois que les ressources financières ont été mobilisées. En effet, il arrive qu'une famille fasse le tour des pharmacies de la ville à la recherche d'un intrant chirurgical/anesthésique.

Le troisième facteur était la durée importante (supérieure à deux heures) de l'intervention chirurgicale. Nous pensons que l'amélioration du plateau technique de l'hôpital (avec mise à disposition de pinces automatiques type agrafeuses linéaires et circulaires, de la thermofusion et des intrants chirurgicaux au bloc opératoire à disposition de l’équipe chirurgicale) pourrait contribuer à diminuer le temps opératoire. Le recyclage des équipes de soins est également une piste d'amélioration.

## Conclusion

L'incidence hospitalière des urgences chirurgicales digestives non traumatiques est importante en Afrique subsaharienne. Les résultats de leur prise en charge chirurgicale ne sont pas satisfaisants en raison d'une morbi-mortalité élevée. Tous les décès dans notre série (10 %) étaient dus à une septicémie ou un choc hypovolémique, soulignant encore le retard diagnostique et l'insuffisance de la prise en charge susévoqués.

Nousavons identifiétrois facteurs modifiables statistiquement associés à un risque accru de complications postopératoires: un délai de consultation supérieur à 72 heures, une prise en charge chirurgicale retardée (supérieure à 48 heures) et une durée d'intervention chirurgicale supérieure à deux heures. La réduction du retard de consultation, la mise sur pied d'un système d'assurance maladie universelle et la réduction du temps opératoire sont des pistes de solution pour améliorer le pronostic de l'urgence chirurgicale digestive non traumatique.

## Lien d'intérêts

Les auteurs ne déclarent aucun lien d'intérêt.

## Source de financement

Aucune.

## Contribution des auteurs

Guy Aristide BANG et Georges BWELLE MOTO: conception de l’étude

Joseph Cyrille CHOPKENG NGOUMFE et Yannick Mahamat EKANI BOUKAR: recueil des données

Fabrice TIENTCHEU TIM et Eric Patrick SAVOM: analyse des données

Guy Aristide BANG et Joseph Cyrille CHOPKENG NGOUMFE: rédaction du manuscrit

Georges BWELLE MOTO, Yannick Mahamat EKANI BOUKAR, Fabrice TIENTCHEU TIM, Eric Patrick SAVOM: relecture et correction du manuscrit

Arthur ESSOMBA: révision finale du manuscrit et autorisation de soumission, caution intellectuelle de l’étude
